# Acute hemolytic transfusion reaction mediated by anti-Jk^b^ antibody in a pediatric patient with β-thalassemia major: a case report illustrating an anamnestic alloimmune response

**DOI:** 10.3389/fimmu.2026.1899658

**Published:** 2026-09-10

**Authors:** Jingyuan Xu, Jian Chen

**Affiliations:** 1 Department of Laboratory Medicine, West China Second University Hospital, Chengdu, China; 2 Department of Transfusion Medicine, West China Second University Hospital, Chengdu, China; 3 Key Laboratory of Birth Defects and Related Diseases of Women and Children, Sichuan University, Ministry of Education, Chengdu, Sichuan, China

**Keywords:** acute hemolytic transfusion reaction, anti-Jk^b^ antibody, Kidd blood group system, pediatric transfusion medicine, β-thalassemia major

## Abstract

Non-ABO alloantibody-mediated acute hemolytic transfusion reactions are infrequent but life-threatening complications of red blood cell transfusion. Anti-Jk^b^ antibodies are difficult to detect in routine pretransfusion screening due to their low titers, weak avidity, and transient persistence. Here, we report a 16-month-old patient with β-thalassemia major who developed AHTR following transfusion of Jk^b^-positive RBCs. The patient had received two prior transfusions without documented adverse reactions. However, following the third transfusion, the patient developed acute intravascular hemolysis within two hours, manifesting as tea-colored urine, fever (39.2 °C), and marked hyperbilirubinemia (total bilirubin 173.7 μmol/L). Hemoglobin declined from 62 g/L to 28 g/L over three days. Serologic workup revealed a positive direct antiglobulin test (1+), and antibody screening showed weak agglutination. Serologic workup confirmed the presence of anti-Jk^b^ antibody. Further SCL14A1 genetic testing confirmed the absence of Jk^b^ gene expression, and the patient’s Kidd phenotype was determined as Jk (a+wb-). Subsequent transfusion of Jk^b^-negative, antigen-matched RBCs caused no hemolytic manifestations, and follow-up revealed that the patient’s hemoglobin remained stable at approximately 90g/L following red blood cell transfusion. This case highlights the diagnostic challenges associated with anti-Jk^b^ alloantibodies and illustrates the clinical features of an anamnestic alloimmune response. These findings underscore the importance of rigorous antibody screening and antigen-matched transfusion in chronically transfused patients with thalassemia.

## Introduction

Acute hemolytic transfusion reactions (AHTRs) are among the most serious complications of RBC transfusion, typically occurring within 24 hours of transfusion. While ABO incompatibility remains the most common cause of severe AHTRs, unexpected antibodies directed against non-ABO blood group antigens can also precipitate clinically significant hemolytic reactions (1, 2). The Kidd (JK) blood group system discovered in 1951 contains three antigens: Jk^a^, Jk^b^, and Jk3. The JK glycoprotein is encoded by the SLC14A1 gene on chromosome 18, functions as a urea transporter in the erythrocyte membrane, and is also expressed on renal endothelial cells (3).

Anti-JK antibodies have been reported to cause both delayed and acute hemolytic transfusion reactions (4). Anti-JK antibodies are typically transient. They are difficult to detect in routine laboratory tests. These antibodies show low titers, weak affinity, and dose-dependent reactivity *in vitro*. After immunologic stimulation, their concentrations decline rapidly. Thus, previously sensitized individuals often test serologically negative on subsequent screening (5). These properties frequently result in false-negative antibody screening outcomes. Antibodies against JK antigens are particularly problematic because they elicit robust anamnestic immune responses, leading to delayed hemolytic transfusion reactions.

β-Thalassemia major is an inherited hemoglobinopathy characterized by ineffective erythropoiesis and severe chronic hemolytic anemia. Patients require lifelong regular transfusion to sustain survival and normal growth (6). These patients face a markedly elevated alloimmunization risk relative to typical transfusion recipients due to repeated allogeneic RBC antigen exposure (7). Furthermore, Kidd blood group incompatibility can cause hemolytic disease of the fetus and newborn (HDFN), and when coexistent with other etiologies of fetal anemia (such as thalassemia) may produce a synergistic effect exacerbating the severity of fetal anemia (8). These findings indicate that Kidd blood group antibodies are not uncommon in chronically transfused populations such as thalassemia patients. Anti-JK antibodies exhibit occult immunologic characteristics. These antibodies may cause severe clinical consequences. Enhanced pretransfusion antibody screening and compatibility testing are therefore clinically important in these patients. Herein we report a rare case of AHTR triggered by anti-Jk^b^ antibody in a pediatric patient with β-thalassemia major, highlighting the clinical significance and diagnostic challenges associated with Kidd system antibodies in transfusion-dependent patients.

## Case report

A 16-month-old patient with β-thalassemia major was admitted to the Emergency Pediatrics Department at West China Second University Hospital, presenting with gross hematuria, fever, and pallor two hours after transfusing 1 unit of leukocyte-reduced red blood cell suspension (LR-RBCs). His mother was identified as a carrier of β-thalassemia trait. The results of β-globin gene testing of the patient and his father showed that both of them were Chinese type Gγ+(Aγδβ) 0 thalassemia heterozygote, and the genotype of the child was Chinese type deletion (Gγ+(Aγδβ) 0) combined with CD41–42 double heterozygous cross. There was no history of consanguineous marriage or other hereditary disorders in the family. Since diagnosis, the patient had received regular transfusion therapy, with two prior documented transfusions without recorded adverse reactions.

**Figure 1:**
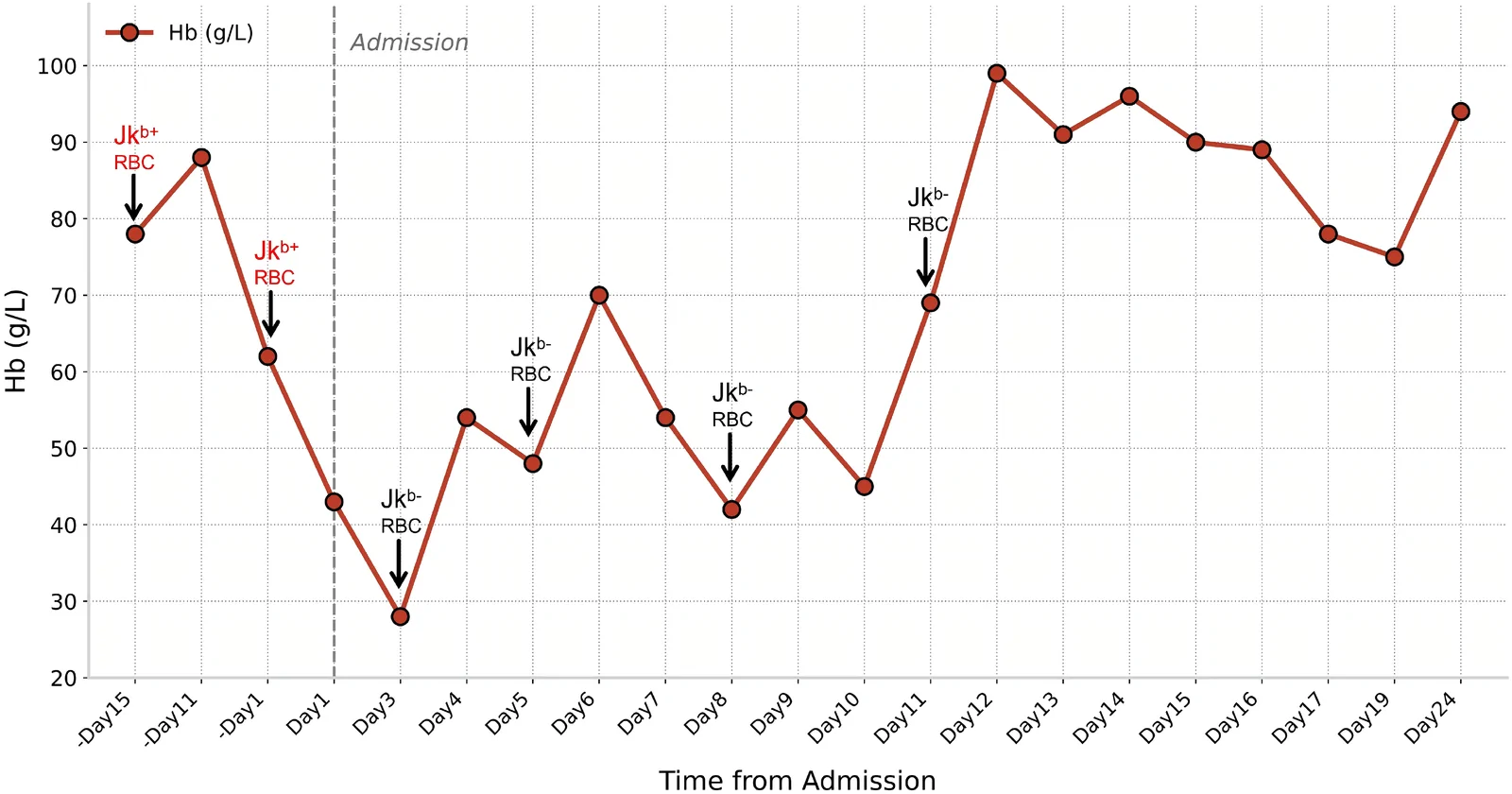
Laboratory test results of Hemoglobin (Hb) in patient. Jk^b+^ RBC indicates transfuse 1 unit of red blood cells that express Jk^b^ antigen, Jk^b-^ RBC indicates transfuse 1unit of red blood cells that did not express Jk^b^ antigen.

Review of the patient’s transfusion history revealed the following: Fifteen days before admission, the patient received the first transfusion of 1unit LR-RBCs (O^+^, Jk^b+^). The pretransfusion hemoglobin level was 63 g/L, increasing to 78 g/L post-transfusion. The direct antiglobulin test (DAT) was negative, and no adverse reactions were observed. Ten days before admission, the patient received a second transfusion of 1unit Jk^b-^ LR-RBCs. The transfusion was uneventful, and no post-transfusion laboratory investigations were performed. The day before admission, the patient received a third transfusion of 1unit LR-RBCs (O^+^, Jk^b+^). Two hours after transfusion, the patient developed the following signs and symptoms: tea-colored urine (gross hematuria), fever (maximum body temperature 39.2 °C) and pallor. Laboratory findings demonstrated: urine occult blood (3+), total bilirubin 173.7 μmol/L (reference range: 5-23 μmol/L), direct bilirubin 9.9 μmol/L (reference range: <8 μmol/L), indirect bilirubin 163.8 μmol/L (reference range: <17 μmol/L), post-transfusion hemoglobin 62 g/L, reticulocyte count 2.06%, hemoglobin declined to 43 g/L on the following day, DAT positive (1+), antibody screening: I (0), II (W), III (0) (Diagnostic Grifols). Antibody identification using column agglutination technology confirmed the presence of anti-Jk^b^ antibody in the patient’s plasma (**Table 1**), and the genotype of the Kidd blood group system determined as Jk(a+wb-). During hospitalization, the patient had a history of ceftriaxone administration. Given that hemoglobin levels decreased to 28 g/L on hospital day 3, we initially suspected drug-induced immune hemolysis. However, drug antibody testing showed negative results (**Supplementary Figure 4**), thereby excluding drug-mediated hemolysis. On hospital day 3, the patient was transfused with 1unit Jk^b^-negative LR-RBCs (O^+^, Jk^b-^), which was well tolerated, with post-transfusion hemoglobin rising to 54 g/L. DAT remained weakly positive (1+), and antibody screening showed I (0), II (W), III (0). On hospital day 5, hemoglobin decreased to 48 g/L, and the patient received 1unit Jk^b-^ LR-RBCs, with hemoglobin increasing to 70 g/L post-transfusion, confirming transfusion efficacy. During this period, therapeutic interventions were primarily supportive management for hemolytic anemia: methylprednisolone for anti-inflammatory effect, hydration and urinary alkalinization, ceftriaxone for infection prophylaxis, intravenous immunoglobulin for antibody neutralization, and corticosteroids for immunosuppression (More details about medications are provided in **Supplementary Table 2**). On hospital day 8, hemoglobin again decreased to 42 g/L, physical examination revealed hepatosplenomegaly (liver palpable 11 cm below the costal margin; spleen: Hodge’s line I 14 cm, line II 11 cm, line III + 2.5 cm). The treating physicians attributed the hemoglobin decline to hypersplenism-mediated ongoing RBC destruction. Consequently, the patient received 1unit Jk^b-^ LR-RBCs, followed by 2 units Jk^b-^ LR-RBCs on hospital day 11. Hemoglobin peaked at 99 g/L and subsequently stabilized at approximately 90 g/L. With resolution of hemolysis, the patient was discharged on hospital day 24. **Table 2** and **Supplementary Table 1** shows the summary of patients’ blood transfusion and effects after transfusion. **Figure 1** shows the hemoglobin results of the patient. More laboratory results are shown in **Supplementary Figures 1–3**.

**Table 1:** Antibody Reaction Pattern of Patient Plasma Specimen Following Acute Hemolytic Transfusion Reaction.

Cell	Rh-Hr								Kell						Duffy		Kidd		Lewis		P	MNS				Luther		Xg	
	C	D	E	c	e	C^w^	f	V	K	k	Kp^a^	Kp^b^	Js^a^	Js^b^	Fy^a^	Fy^b^	Jk^a^	Jk^b^	Le^a^	Le^b^	P1	M	N	S	s	Lu^a^	Lu^b^	Xg^a^	Patient plasma reaction pattern
1	+	+	0	0	+	+	/	/	0	+	0	+	/	+	+	0	+	0	0	+	+	+	+	+	0	0	+	+	0
2	+	+	0	0	+	0	/	/	0	+	0	+	/	+	0	+	+	0	+	0	+	0	+	0	+	0	+	/	0
3	0	+	+	+	0	0	/	/	0	+	0	+	/	+	+	+	+	+	+	0	0	+	0	0	+	0	+	+	w
4	0	+	0	+	+	0	/	/	0	+	0	+	/	/	0	+	0	+	0	+	0	+	+	0	+	0	+	/	w
5	+	0	0	0	+	0	/	/	0	+	0	+	/	+	+	+	+	+	0	+	+	+	0	+	+	0	+	+	1+
6	0	0	+	+	0	0	/	/	0	+	0	+	/	+	0	+	+	0	0	+	+	+	+	0	+	0	+	+	w
7	0	0	0	+	+	0	/	/	+	0	0	+	/	+	0	+	+	+	0	+	0	+	0	+	0	0	+	0	w
8	0	0	0	+	+	0	/	/	0	+	+	0	0	+	0	+	0	+	+	0	+	+	+	0	+	0	+	+	1+
9	0	0	0	+	+	0	/	/	+	+	0	+	0	+	+	0	+	0	+	0	+	0	+	0	+	0	+	+	0
10	0	0	0	+	+	0	/	/	+	+	0	+	0	+	0	+	+	+	+	0	+	+	+	+	+	0	+	+	0
11	+	+	+	0	+	0	/	/	+	+	0	+	/	+	+	+	0	+	0	+	0	+	+	+	+	0	+	+	w
12	w	+	+	+	0	0	/	/	0	+	+	+	/	+	+	0	+	+	0	+	+	+	+	+	+	0	+	+	w
13	0	0	0	+	+	0	/	/	0	+	0	+	/	+	+	+	+	0	0	0	0	0	+	0	+	0	+	+	0
14	+	+	+	+	+	0	/	/	0	+	0	+	/	+	+	0	0	+	+	0	+	+	+	0	+	+	+	0	1+
15	+	+	0	0	+	0	/	/	+	+	0	+	/	+	+	0	+	0	0	+	+	+	0	+	0	0	+	0	0
16	0	0	0	+	+	0	/	/	0	+	0	+	/	+	+	+	+	0	0	+	+	+	+	+	+	0	+	/	0

“+” indicates a positive reaction; higher values indicate stronger agglutination intensity; “0” indicates a negative reaction; “/” indicates not tested; “w” indicates a weak positive reaction.

**Table 2:** Summary of patients’ blood transfusion and effects after transfusion.

Time from admission	Donor blood type	Transfusion dose	Compatibility testing	Results and reactions
-Day15	O^+^ Jk^b+^	1U	Major crossmatch: Compatible Minor crossmatch: Compatible	Effective transfusion
-Day10	O^+^ Jk^b-^	1U	Major crossmatch: Compatible Minor crossmatch: Compatible	No Follow-Up test
-Day1	O^+^ Jk^b+^	1U	Major crossmatch: Compatible Minor crossmatch: Compatible	Acute Hemolytic Reaction
				DAT (1+), Antibody Screen: I (0) II(w) III (0)
				Antibody identification: Anti-Jk^b^
				Kidd Phenotype: Jk(a+wb−)
Day 3	O^+^ Jk^b-^	1U	Major crossmatch: Compatible Minor crossmatch: Incompatible	Effective transfusion
				DAT (1+), Acid Elution: Negative, Antibody Screen: I (0) II(w) III (0)
				Specimen Ceftriaxone Drug Antibody: Negative
Day 5	O^+^ Jk^b-^	1U	Major crossmatch: Compatible Minor crossmatch: Incompatible	Effective transfusion
				Acid Elution: Negative, Antibody Screen: I (0) II(w) III (0)
				DAT(w)
Day 8	O^+^ Jk^b-^	1U	Major crossmatch: Compatible Minor crossmatch: Incompatible	Effective transfusion
				Specimen Acid Elution: Negative, DAT(w)
Day 11	O^+^ Jk^b-^	2U	Major crossmatch: Compatible Minor crossmatch: Incompatible	No Adverse Reaction, effective transfusion

Day1= The first day of admission; O^+^ Jk^b-^ indicates type O, RhD positive, Jk^b^ negative Leukocyte-reduced red blood cell suspension; O^+^ Jk^b+^ indicates type O, RhD positive, Jk^b^ positive Leukocyte-reduced red blood cell suspension; “+” indicates a positive reaction; higher values indicate stronger agglutination intensity; “w” indicates a weak positive reaction.

At our institution, routine pretransfusion testing includes ABO and RhD typing, antibody screening, and cross-matching using column agglutination technology. Extended antibody identification and red cell phenotyping for clinically relevant antigens are performed when irregular antibodies are detected or upon physician request. Anti-Jk^b^ was identified in this patient. All subsequent transfusions used Jk^b^-negative, cross-match-compatible red blood cells. In the coming month, the patient received three separate infusions of 1unit Jk^b-^ LR-RBCs without any transfusion reaction. The latest follow-up results showed that, the hemoglobin level was 90 g/L, and both the DAT and the antibody screen were negative. We have established a dedicated blood transfusion record for this patient with Jk^b-^ red blood cells, marked with the warning label “Anti-Jk^b^ antibody previously detected in plasma, Jk^b-^ RBC must be transfused”. The patient was enrolled in the special transfusion management program. Donor red blood cells must be screened for the Jk^b^ antigen before each transfusion. Long-term follow-up will be performed to monitor the patient’s outcome.

## Discussion

From an immunological perspective, initial alloimmunization to red blood cell antigens activates naïve B cells. This process generates memory B cells that persist for years. Upon re-exposure to the same antigen, these memory B cells rapidly differentiate into antibody-secreting cells (9). The resulting secondary immune response is characterized by accelerated kinetics and increased antibody affinity. Pre-formed or anamnestic antibodies may then bind to donor RBC antigens. This antigen-antibody interaction activates the complement cascade. Complement activation subsequently causes intravascular hemolysis and massive release of inflammatory mediators, culminating in AHTR. Circulating antibodies are secreted by plasma cells, but they do not confer immunological memory. Once transfused red blood cells are cleared, antibody levels gradually decline. This decline results from the absence of sustained antigenic stimulation, the short lifespan of plasma cells, and the physiological catabolism of IgG (10, 11). Eventually, antibody titers may fall below the detection limit of serological assays.

Notably, Kidd antibodies are among the alloantibodies most prone to evanescence, with reported disappearance rates of 40% to 60% (11). Despite negative serological results, memory B cells can persist for long periods and mount a rapid recall response upon re-exposure to the Jk^b^ antigen. This mechanism highlights the importance of maintaining complete transfusion records and transfusing antigen-negative blood, even when the antibody is no longer detectable (12, 13). In this case, the hemolysis of this patient developed rapidly within two hours, hemoglobin levels fell markedly, DAT yielded positive results. These observations are consistent with a brisk IgG-mediated process involving complement activation. Serologic detection of Kidd blood group system antibodies remains a challenge of transfusion medicine. These antibodies commonly exhibit low titers, weak affinity, and dose-dependent reactivity. They typically disappear rapidly following initial immunization. In previously sensitized individuals, subsequent testing may yield false-negative results, creating a misleading “safety illusion” in pretransfusion compatibility testing. As demonstrated in this case and previous reports, antibody detection may require multiple transfusion episodes before becoming apparent. Antibodies of the Kidd blood group system frequently cause hemolytic transfusion reactions. Among clinically significant non-ABO antibodies, anti-Jk^a^ and anti-Jk^b^ account for a considerable proportion (3). A meta-analysis demonstrated that the detection rate of Kidd blood group system associated RBC alloantibodies among transfusion-dependent thalassemia patients in mainland China is approximately 6.5% (14). Anti-Jk^a^ is more common than anti-Jk^b^. It can cause both DHTR and AHTR (15, 16), in contrast, AHTR mediated by anti-Jk^b^ antibody is exceedingly rare (5, 17), this case represents the first report of anti-Jk^b^ mediated AHTR in a pediatric patient. Notably, when routine DAT complement testing yields negative results, flow cytometry may serve as a valuable adjunctive tool for detecting anti-Jk^b^ IgM and holds significant clinical utility (17),

Patients with severe β-thalassemia require lifelong red blood cell transfusion. Repeated exposure to allogeneic antigens places them at high risk for alloimmunization. Therefore, enhanced transfusion safety measures are essential for these patients. Red blood cell genotyping offers unique advantages for transfusion-dependent patients. It provides a comprehensive antigen profile. Furthermore, it is not affected by recent transfusions or alloimmunization. Post-transfusion fever, hemoglobinuria, or unexplained worsening of anemia should raise suspicion of AHTR. This suspicion remains valid even if pre-transfusion antibody screening was negative. Immediate serological investigations are required. These include monospecific DAT, antibody identification, and cross-matching. Long-term management of patients with anti-Jk^b^ requires close collaboration between clinicians and transfusion medicine specialists. This ensures timely access to Jk^b-^ blood products. Subsequent transfusion decisions should be based on careful risk-benefit assessment. A sufficient inventory of Jk^b-^, crossmatch-compatible donor units must also be secured.

Current guidelines advocate extended red cell antigen typing before the first transfusion in patients with β-thalassemia. The ISBT recommends phenotyping or genotyping for Rh (D, C, c, E, e) and Kell antigens in all β-thalassemia patients prior to their first transfusion, ideally before 1 year of age. Similarly, both the AABB and the TIF guidelines (18) recommend red cell genotyping or extended phenotyping for patients anticipated to require chronic transfusion support, with matched transfusion for Rh (D, C, c, E, e) and K antigens whenever full phenotyping or genotyping is available. Taken together, extended antigen typing for Rh (D, C, c, E, e) and Kell should be performed before transfusion initiation, and early implementation is essential for preventing alloimmunization in patients requiring lifelong transfusion therapy.

It is worth mentioning that this study has several limitations. First, we could not definitively confirm that this case was a secondary anamnestic immune response as antibody screening was not performed immediately after the first transfusion, and memory B-cell studies were not conducted. Therefore, the classification of this case as an anamnestic response was based on the clinical presentation and the patient’s partial serological findings, rather than on direct immunological evidence. Secondly, we did not perform immunoglobulin subclass determination or complement subtype analysis. This limited the mechanistic depth of the immunological investigation.

## Conclusion

We report a rare case of AHTR mediated by anti-Jk^b^ antibody in a pediatric patient with β-thalassemia major. The diagnosis was established through clinical evaluation and serologic investigation and confirmed by successful transfusion of Jk^b-^ RBCs without recurrence of hemolytic manifestations. This case highlights the diagnostic challenges associated with Kidd system antibodies, which are characterized by low titer, transient expression, and propensity to cause false-negative screening results. Clinicians and blood bank professionals must maintain a high index of suspicion for hemolytic reactions in multiply transfused patients and ensure implementation of comprehensive antibody screening protocols. Enhanced awareness of the clinical significance of non-ABO alloantibodies, particularly in transfusion-dependent populations such as thalassemia patients, is essential for improving transfusion safety and patient outcomes.

## Data Availability

The original contributions presented in the study are included in the article/**Supplementary Material**. Further inquiries can be directed to the corresponding author.
