# Supplementary material for: Acute hemolytic transfusion reaction mediated by anti-Jkb antibody in a pediatric patient with β-thalassemia major: a case report illustrating an anamnestic alloimmune response
Source: Front Immunol. 2026 Sep 10;17:1899658. doi: 10.3389/fimmu.2026.1899658 (PMC13600969; doi:10.3389/fimmu.2026.1899658)
Supplement: Supplementary file 1 [file SupplementaryFile1.docx]

| Date | Admission time | U-OB | Urine color | urobilinogen | antibody screening | Antibody titration | Self control | DAT | | | Acid elution test |
| --- | --- | --- | --- | --- | --- | --- | --- | --- | --- | --- | --- |
|  |  |  |  |  |  |  |  | IgG | IgM | C3d |  |
| April 16, 2026 | -Day18 | weak | yellow | negative | negative | / | / | / | / | / | / |
| April 19, 2026 | -Day15 | / | / | / | negative | / | negative | negative | / | / | / |
| April 23, 2026 | -Day11 | / | / | / | negative | / | negative | negative | / | / | / |
| May 3, 2026 | -Day1 | / | / | / | negative | / | negative | negative | / | / | / |
| May 4, 2026 | Day1 | 3+ | red | negative | I(0)II(w)III(0) | ＜1 | weak | Positive  (1+) | negative | negative | / |
| May 6, 2026 | Day3 | / | / | / | I(0)II(w)III(0) | ＜1 | weak | Positive  (1+) | negative | negative | Negative |
| May 10, 2026 | Day7 | 3+ | yellow | 3+ | I(w)II(2+)III(2+) (PEG) | 1 | 2+ (PEG) | Positive (1+) | negative | negative | Negative |
| May 12, 2026 | Day9 | / | / | / | I(0)II(1+)III(0) | 1 | weak | Positive (2+) | negative | negative | / |
| May 13, 2026 | Day12 | / | / | / | I(0)II(1+)III(0) | 1 | weak | Positive (1+) | negative | negative | / |

Supplement Table 1. Other laboratory results of the patient

Supplement Table 2. Patient medication

|  | Furosemide | Gamma globulin | Methylprednisolone | Ceftriaxone |
| --- | --- | --- | --- | --- |
| Day1 | / | 10g | 56mg | 0.8g |
| Day2 | / | 10g | 40mg | 1g |
| Day3 | / | / | 80mg | 1g |
| Day4 | 8mg | / | 40mg | 1g |
| Day5 | / | / | 120mg | 1g |
| Day6 | 5mg | / | 40mg | / |
| Day7 | 5mg | / | 100mg | / |
| Day8 | 5mg | / | 30mg | / |
| Day9 | 5mg | / | 100mg | / |
| Day10 | 5mg | 10g | 100mg | / |
| Day11 | 10mg | 10g | 50mg | / |
| Day12 | 5mg | / | 50mg | / |
| Day13 | / | / | 20mg | / |
| Day14 | / | / | 20mg | / |
| Day15 | / | / | 10mg | / |

Supplement Figure 1. Platelet Count (×10^9^/L)

Supplement Figure 2. Bilirubin (umol/L)

Supplement Figure 3. Percentage of reticulocytes (%)

Patient plasma+Ceftriaxone (-Day1)

Patient plasma+PBS (-Day1)

Patient plasma+Ceftriaxone (Day3)

Patient plasma+PBS (Day3)

control plasma+Ceftriaxone

control plasma+PBS

Supplement Figure 4. Drug antibody test results
